# Mumps Virus: Modification of the Identify-Isolate-Inform Tool for Frontline Healthcare Providers

**DOI:** 10.5811/westjem.2016.6.30793

**Published:** 2016-06-30

**Authors:** Kristi L. Koenig, Siri Shastry, Bandr Mzahim, Abdulmajeed Almadhyan, Michael J. Burns

**Affiliations:** * University of California Irvine, Medical Center, Department of Emergency Medicine, Center for Disaster Medical Sciences, Orange, California; † University of California Irvine, Medical Center, Department of Emergency Medicine, and Department of Medicine, Division of Infectious Diseases, Orange, California; ‡ University of California Irvine Medical Center, Department of Emergency Medicine, Orange California; § Qassim University, Department of Emergency Medicine, Saudi Arabia; ¶ King Fahad Medical City, Department of Emergency Medicine, Saudi Arabia

## Abstract

Mumps is a highly contagious viral infection that became rare in most industrialized countries following the introduction of measles-mumps-rubella (MMR) vaccine in 1967. The disease, however, has been re-emerging with several outbreaks over the past decade. Many clinicians have never seen a case of mumps. To assist frontline healthcare providers with detecting potential cases and initiating critical actions, investigators modified the “Identify-Isolate-Inform” tool for mumps infection. The tool is applicable to regions with rare incidences or local outbreaks, especially seen in college students, as well as globally in areas where vaccination is less common. Mumps begins with a prodrome of low-grade fever, myalgias and malaise/anorexia, followed by development of nonsuppurative parotitis, which is the pathognomonic finding associated with acute mumps infection. Orchitis and meningitis are the two most common serious complications, with hearing loss and infertility occurring rarely. Providers should consider mumps in patients with exposure to a known case or international travel to endemic regions who present with consistent signs and symptoms. If mumps is suspected, healthcare providers must immediately implement standard and droplet precautions and notify the local health department and hospital infection control personnel.

## INTRODUCTION

Several international public health crises have emerged in recent years, including Ebola, Middle East respiratory syndrome (MERS), and Zika virus. In addition to these novel threats, there has been a resurgence of previously nearly eradicated infectious diseases, including mumps. In recent years, the numbers of mumps cases in the United States has fluctuated from hundreds to thousands of cases per year. In 2006, a multi-state mumps outbreak in the Midwest consisted of over 6,500 cases. In 2009–2010, two large outbreaks occurred in New York City and Guam, affecting about 3,000 and 500 persons respectively. In 2011–2013, there were smaller outbreaks in several states.^1^ Many of the outbreaks occurred among college students. There was also a large outbreak in late 2014 among professional hockey players involving at least five teams in the National Hockey League (NHL), which began with players on the Anaheim Ducks. In March 2016, California public health officials issued an advisory noting that five college students at the University of San Diego had been diagnosed with mumps; this was followed by a subsequent advisory identifying three additional mumps cases in college students diagnosed in Orange County, California.^2^,^3^ In April 2016, a high profile outbreak reported at Harvard University and surrounding areas resulted in more than 40 cases of mumps in less than two months. As of May 5, 2016, nearly 80 cases were reported in the state of Massachusetts with 50 cases at Harvard.^4^ Over a 5-year period from 2011 to 2016, mumps cases reported to the Centers for Disease Control and Prevention (CDC) have been steadily increasing, from 370 in 2011 to 1,148 as of May 21, 2016.^5^ These cases and outbreaks as well as the potential decrease in measles-mumps-rubella (MMR) vaccination uptake due to parental refusal underscore the importance of the emergency department (ED) as a primary location for identification and containment of public health threats. Patients frequently present to the ED with undifferentiated chief complaints, making rapid and accurate diagnosis challenging. Most contagious diseases that manifest with nonspecific influenza-like illness symptoms and signs will not ultimately be determined to be rare and deadly diseases like Ebola or MERS; however, they may be contagious and require immediate isolation. This underscores the need for emergency physicians to have the necessary information and tools to rapidly identify potential public health threats. While the mumps virus is typically mild and self-limited, it is highly contagious for susceptible patients when proper isolation and containment measures are not rapidly initiated; a single case can result in up to 12 secondary cases in a susceptible population.^6^ Infection can occur despite vaccination, and most cases seen in college outbreaks have occurred in fully vaccinated patients. Further, the mumps virus can sometimes have serious long-term sequelae including infertility/subfertility, central nervous system (CNS) infection, deafness, and severe pancreatitis. In rare cases, these complications can be fatal.^7^

Given the highly contagious nature of the virus, it is paramount that frontline providers be aware of how to identify the clinical manifestations of mumps virus and understand how to properly isolate potentially infected patients and rapidly inform necessary authorities of a potential case. This paper provides a comprehensive review of mumps infection followed by a brief discussion of the novel 3I tool, initially developed for Ebola virus and subsequently for measles, MERS and Zika virus,^8^–^11^ as adapted for use by healthcare providers in the initial detection and management of mumps.

## CLINICAL PRESENTATION

**Figure 1:**
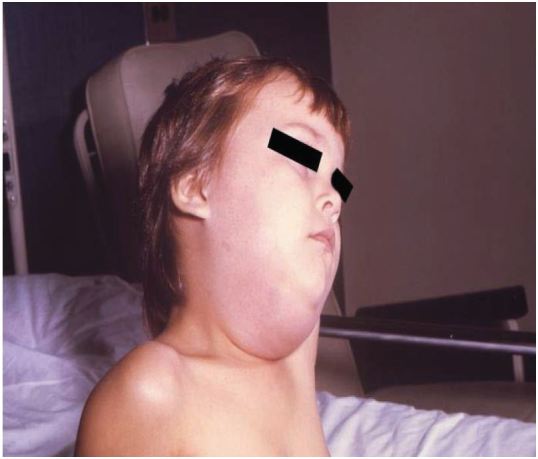
Child with mumps parotitis, the pathognomonic finding of acute mumps infection.

Mumps typically begins with a prodrome of low-grade fever, myalgias, anorexia, malaise and headache. Over the next 1–3 days, the patient develops earache and tenderness over the parotid gland, which becomes noticeably enlarged and painful (Figure 1). Parotitis is typically seen in 31–65% (with some authoritative texts citing 60–70%) of cases of mumps infection. In about three quarters of patients, the other parotid gland becomes involved.^12^,^13^ The parotitis is nonsuppurative and typically progresses for about three days and lasts for approximately one week.^13^ Patients often have trismus and have difficulty chewing and speaking. In about 10% of cases, other salivary glands, especially the submandibular gland, can become involved and can mimic anterior cervical lymphadenopathy.

## RISK FACTORS

In the post-vaccination era, populations at greatest risk for mumps infection are adolescents and adults. The clinical course of mumps also tends to be more severe in adolescents/adults when compared to the clinical course in younger children.^6^ Other at-risk populations include unvaccinated individuals who are exposed to the virus – this includes children whose parents opted against vaccination and those with contraindications to vaccination including anaphylactic/severe allergic reactions to vaccine components or neomycin, and immunocompromised children.^12^ Risk of mumps for travelers is high in many countries, including industrialized countries. For example, the United Kingdom has had several outbreaks since 2004, and Japan does not routinely vaccinate against mumps. The disease is only contracted and spread by humans; there is no animal host.

## DIAGNOSIS

Mumps is a clinical diagnosis that is made based on a history of exposure, prodromal constitutional symptoms and parotitis. Serologic/polymerase chain reaction (PCR) testing to confirm diagnosis is also available. Mumps virus can be isolated from saliva, urine, blood, nasopharyngeal secretions, and seminal fluid.^14^, ^15^ The preferred definitive diagnostic test is a swab of the buccal mucosa using a viral culture swab for RT-PCR testing. Collection of the specimen in the first three days of parotitis is optimal, but virus can still be detected in some cases up to nine days after onset of parotitis. Clinicians should contact local or state public health authorities to arrange for testing, as testing at commercial laboratories may be unreliable. Serologic diagnosis of acute mumps infection by testing for IgM and IgG antibodies may be unreliable, as the IgM response may be attenuated or absent in vaccinated persons, and persons with detectable IgG titers can still develop mumps.^16^

## COMPLICATIONS AND SPECIAL POPULATIONS

In post-pubertal males, the most common complication of mumps infection is orchitis. In the era prior to the advent of the MMR vaccine, orchitis occurred in between 12% to 66% of post-pubertal males with mumps. In the post-vaccination era, orchitis has been reported in 15% to 40% of post-pubertal males.^12^ Orchitis typically occurs about 10 days after the onset of parotitis, although it can be seen up to six weeks later. Orchitis is typically unilateral, but bilateral orchitis manifests in 15–30% of cases.^13^ Orchitis may be accompanied by epididymitis up to 85% of the time.^17^

Mumps orchitis can lead to a range of testicular complications. True infertility following mumps orchitis is rare but subfertility has been seen in up to 13% of patients. Subfertility can occur even without accompanying testicular atrophy.^7^, ^18^–^20^ Testicular atrophy (any reduction in testicular size) occurs in 30–50% of patients with orchitis.^6^ Abnormalities of spermatogenesis have been observed to occur in up to half of patients for up to three months after recovery from the acute illness.^20^ Mumps orchitis and subsequent testicular atrophy have been weakly associated with the development of testicular tumors, including cancer, with an incidence of 0.5%.^7^, ^21^, ^22^

Other complications of mumps infection include meningitis, which may occur in up to 10% of cases. When meningitis does occur, it is typically seen 3–4 days after the onset of parotitis.^6^ Acute encephalitis and encephalomyelitis are rare. When acute encephalitis due to mumps occurs, it is typically self-limiting. Acute encephalomyelitis, on the other hand, tends to be much more severe. Case fatality rates for acute encephalomyelitis due to mumps virus are up to 10%, while the overall case fatality rate due to CNS complications from mumps virus has been reported to be about 1%.^23^

Sensorineural hearing loss is another CNS complication of mumps infection. Permanent unilateral hearing loss has been reported to occur in 1 of every 20,000 cases. Bilateral hearing loss is much less frequent. Other rare CNS complications include Guillain Barre Syndrome, transverse myelitis, facial palsy, cerebellar ataxia and flaccid paralysis.^7^

Oophoritis (ovarian inflammation) has been reported to occur in 5% of post-pubertal females. Symptoms of oophoritis may include lower abdominal pain, vomiting and fever. Long-term sequelae of oophoritis, while rare, may include infertility or premature menopause. Mastitis (breast inflammation) has also been reported as a complication of mumps infection in post-pubertal females.^7^ In some studies, mumps infection in early pregnancy has been linked with spontaneous abortion, with one study identifying a 27% rate of fetal death after first trimester mumps infection compared with 13% in a control group.^7^, ^24^ A second, more recent study has not shown the same association between spontaneous abortion and mumps infection in early pregnancy.^25^ As of early 2016, there is no reported association between perinatal mumps infection and significant congenital malformations.^7^

Other rare complications associated with mumps infection include pancreatitis (with rare reported cases of severe hemorrhagic pancreatitis), ECG abnormalities (depressed ST segments, prolonged PR intervals and inverted T waves), myocarditis, polyarthritis, abnormal renal function (with rare reports of severe or fatal nephritis), hepatitis, acalculous cholecystitis, kerato-uveitis, hemophagocytic syndrome and thrombocytopenia.^7^ (Table)

## TRANSMISSION AND PERSONAL PROTECTIVE EQUIPMENT

Mumps is a moderately to highly contagious infection that is typically transmitted via direct contact, droplet transmission and through spread from contaminated fomites. It is considered to be less contagious than measles or varicella. About one-third of cases are subclinical and these persons are also contagious. The incubation period of mumps virus averages 16–18 days (range 12 to 25 days) and infected patients are most contagious at 1 to 2 days *prior* to symptom onset.^7^, ^26^, ^27^ As of 2008, CDC, the American Academy of Pediatrics (AAP), and the Healthcare Infection Control Practices Advisory Committee (HICPAC) recommend a 5-day period of isolation after the onset of parotitis. Isolation measures should include standard as well as droplet precautions.^28^

## DIFFERENTIAL DIAGNOSIS

The differential diagnosis for mumps includes other causes of parotitis such as Epstein Barr virus, parainfluenza virus types 1 and 3, influenza A virus, coxsackie virus, adenovirus, parvovirus B19, lymphocytic choriomeningitis virus, human immunodeficiency virus (HIV), human herpesvirus 6, and suppurative infection caused by Staphylococcus aureus, gram-negative bacteria and atypical mycobacteria. Non-infectious causes for parotid swelling include starch ingestion, drugs (phenylbutazone, thiouracil, iodides, phenothiazines), malnutrition, tumors, cysts, salivary stones, metabolic disorders (diabetes, cirrhosis, uremia) and rare disorders such as Mikulicz’s, Parinaud’s and Sjogren’s syndromes. Differential diagnosis for mumps orchitis/epididymitis includes bacterial infection and testicular torsion.^13^

## TREATMENT

Mumps virus is typically self-limiting with treatment primarily directed towards supportive care including antipyretics and analgesics. Supportive treatment of mumps orchitis includes bed rest, scrotal support, heat and cold packs as well as antipyretics and analgesics. Antibiotics are also commonly prescribed both because it can be difficult to distinguish mumps orchitis from bacterial infection and to prevent superimposed bacterial infection.^29^, ^30^

Treatment with mumps intramuscular immunoglobulin has been shown to have no benefit in mumps epidemics, although the immunoglobulin may have some benefit in early infection in a limited number of cases.^31^, ^32^ Although intravenous immunoglobulin may reduce some complications of mumps, there is no universal recommendation for its use.^7^

Historically, various methods to reduce intratesticular pressure, including interferon therapy,^33^–^35^ treatment with oxyphenbutazone^36^ and surgical management including aspiration^37^, ^38^ have been described. Results of these treatments have been variable with unclear impact on the development of long-term testicular atrophy and other complications. Accordingly, there is no universal recommendation in favor of these measures and they are not commonly used in clinical practice.^13^

## PREVENTION

The primary method of prevention of mumps infection is via vaccination. Current vaccination recommendations are for a two-dose vaccination series for all children with the MMR vaccine. The first dose of the vaccine should be administered at age 12–15 months and the second dose of the vaccine should be given at 4–6 years of age. Previously unvaccinated school-aged children/post high school students, international travelers and healthcare providers should also receive two doses of the MMR vaccine while other unvaccinated adults should receive one dose of the vaccine. CDC estimates that about 90% of people are protected after two MMR doses. Like other live virus vaccines, the MMR vaccine should not be administered to pregnant women, persons on immunosuppressive therapy, those with congenital or acquired immunodeficiency disorders, persons with severe febrile illnesses, advanced malignancies, or those persons with advanced HIV disease. Other prevention methods include isolation of cases as detailed previously as well as use of respiratory hygiene/cough etiquette.^28^

## DISPOSITION

Hospital admission is not typically indicated for mumps infection except for cases of serious neurologic and other CNS sequelae, other severe complications, or in cases where patients meet standard hospital admission criteria for other conditions, or require aggressive supportive care measures. In practice, hospitalization for mumps is uncommon.^28^

## IDENTITY-ISOLATE-INFORM

**Figure 2:**
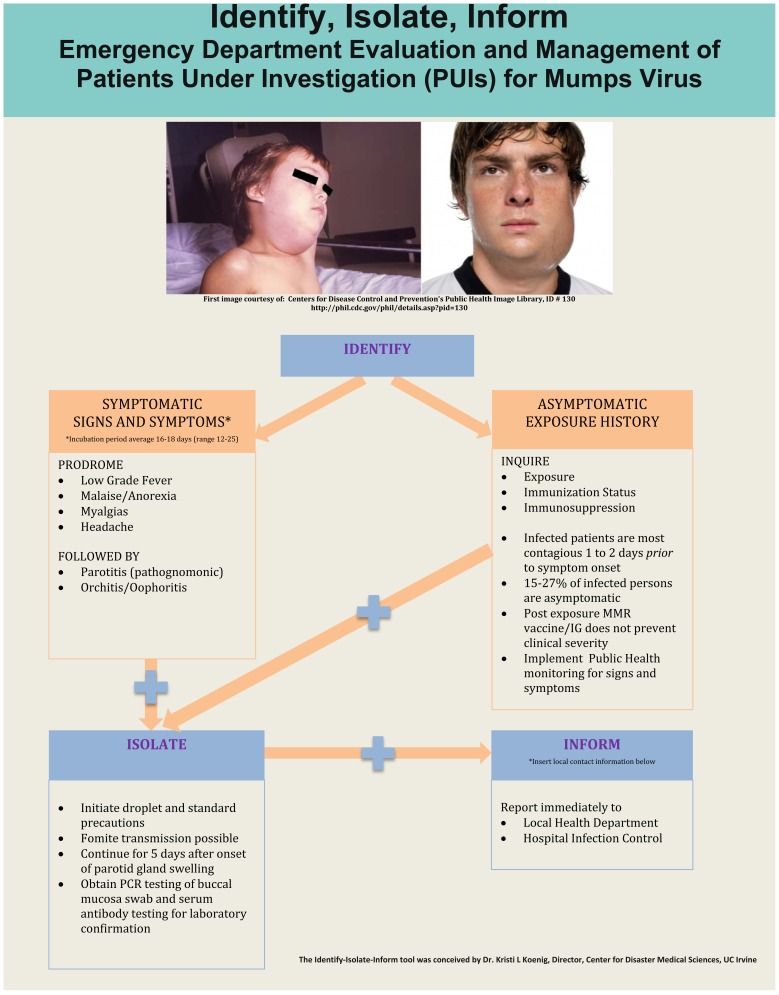
Identify-Isolate-Inform Tool adapted for mumps virus.

The Identify-Isolate-Inform (3I) tool initially developed for Ebola virus disease^8^ can be modified for ED evaluation and management of patients under investigation for mumps (Figure 2). The 3I tool was conceived during the 2014 Ebola virus disease outbreak as a concise method to identify and manage patients presenting to the ED who might have Ebola. The first step was to identify patients with an epidemiologic risk factor (potential exposure to a symptomatic Ebola patient) coupled with symptoms of disease. Once identified as a “patient under investigation,” isolation had to be immediately implemented and both public health and hospital infection prevention authorities notified of the case. The 3I tool was conceived by Koenig, approved by the American College of Emergency Physicians Expert Ebola Panel and adopted and distributed to EDs nationwide by the CDC. Subsequently the 3I tool was modified for use in MERS, measles, and Zika viruses, in each case considering disease characteristics (e.g., contagious prior to or only after symptom onset, incubation periods, epidemiologic risk factors, types of isolation necessary).

As with any patient presenting to the ED, the “vital sign zero” concept should be applied immediately to determine whether the patient is a potential threat to healthcare providers or other patients.^39^ This means that prior to touching a patient to measure the traditional vital signs, triage nurses must consider whether the patient may be contagious (or contaminated with a risk of contaminating others) and don disease/contaminant-characteristic specific personal protective equipment (PPE) and initiate appropriate isolation measures to protect healthcare providers and other patients/visitors from contagion/contaminant. While the specific diagnosis may often be unknown at initial presentation, certain epidemiologic risk factors (e.g., recent travel to West Africa for Ebola virus disease) or other risk factors (e.g., close contact with a known asymptomatic infected patient in the case of measles) coupled with clinical features (e.g., parotid swelling in the case of mumps) should lead to the immediate placement of a person into the “patients under investigation” category.

The majority of patients with mumps infection will likely present to the ED with symptoms; however, it is also possible that asymptomatic patients will present during an outbreak due to concerns about potential exposure to the virus. Accordingly, the first branch of the algorithm involves determination of whether the patient is symptomatic or asymptomatic. For asymptomatic patients, exposure history must be elicited as mumps can be transmitted 1–2 days prior to symptom onset. For patients with a history of exposure to the mumps virus, there is no clear evidence to support reduction of clinical disease severity through post-exposure MMR vaccination or immunoglobulin administration.^7^ Public health monitoring of asymptomatic patients for development of signs and symptoms is recommended. Each prior 3I tool (e.g., for Ebola, MERS, measles, and Zika) considered specific disease characteristics, and the algorithms were modified accordingly. For example, Ebola is not contagious from person to person prior to symptom onset, and therefore, no PPE or isolation would be indicated in an asymptomatic person.

In the case of mumps, symptomatic patients who exhibit either the viral prodrome (low grade fever, myalgias, anorexia, headache) after a known exposure to mumps or the hallmark finding of parotitis, should immediately be masked and isolated using droplet and standard precautions.^28^ Healthcare providers should elicit exposure history, vaccination status and medical history to determine whether the affected patient is immunocompromised. All healthcare providers, regardless of vaccination status, should wear a gown, gloves and a mask when caring for these patients. Isolation precautions should be continued for five days after the onset of parotid gland swelling to minimize risk of disease transmission. Laboratory specimens (serum, urine, nasopharyngeal secretions, semen) should be sent for confirmatory PCR and serologic testing.

Healthcare providers should promptly notify public health authorities about suspected mumps cases or suspected cases of asymptomatic exposure. Providers should also inform hospital infection control of the suspected case and/or exposure.

## LIMITATIONS

While strengths of the tool include that it is concise and can be made readily available, frontline clinicians must suspect mumps so that they can then apply the tool. This may be particularly challenging since viral prodromes are a common presentation in ED patients and resources are insufficient to isolate all such patients. Hence, inquiring about exposure as appropriate (e.g., during a known outbreak) and being aware of the classic clinical appearance of parotid swelling (even without a known outbreak) is crucial.

This study is further limited in that it represents the derivation of the mumps 3I tool and the tool has not been widely validated under real-time conditions. In addition, the 3I tool is generic and we recommend that it be populated with the local 24/7 contact numbers for public health and hospital infection prevention as it may not be simple to rapidly identify contact information, particularly in the off hours.

## CONCLUSION

Mumps is a highly contagious viral disease that became rare following implementation of the MMR vaccination but has been reemerging in the last decade with multiple outbreaks of hundreds to thousands of cases per year, often in college students living under crowded conditions. Undifferentiated patients presenting to the ED with influenza-like illness may have a myriad of diseases with variable characteristics; parotid swelling is pathognomic for mumps. Mumps is a viral illness that is contagious from person to person prior to symptom onset and can be readily transmitted if not identified so that proper precautions can be initiated. Identify-Isolate-Inform is a useful tool for emergency physicians to apply in the evaluation and management of patients with possible mumps infection who present to the ED.

## Figures and Tables

**Table:** Complications associated with mumps infection.

Complication	Frequency
Orchitis	15–40% of postpubertal males with infection
Epididymitis	Accompanies up to 85% of cases of orchitis
Bilateral orchitis	15–30% of epididymitis or orchitis cases
Subfertility	Up to 13% of male patients
Testicular atrophy	30–50% of patients with orchitis
Testicular tumors	0.5% of cases of mumps orchitis or testicular atrophy
Oophoritis	5% of postpubertal females with infection
Meningitis	1–10% of infections
Encephalitis	0.1% of infections
Death due to CNS complications of mumps	1 to 1.5% of cases with CNS complications
Permanent unilateral hearing loss	0.005% of infections
Spontaneous abortion	27% of pregnancies with mumps infection in the first trimester
Pancreatitis	4% of infections

*CNS,* central nervous system
